# ASO Author Reflections: Nationwide Reflection of Clinical Outcomes in Elderly Patients after Resection of Pancreatic Cancer

**DOI:** 10.1245/s10434-022-11920-7

**Published:** 2022-05-26

**Authors:** Anne Claire Henry, Thijs J. Schouten, Lois A. Daamen, I. Quintus Molenaar, Hjalmar C. van Santvoort

**Affiliations:** Department of Surgery, Regional Academic Cancer Center Utrecht, UMC Utrecht Cancer Center & St. Antonius Hospital Nieuwegein, Utrecht, The Netherlands

## Past

Over the years, resection of pancreatic cancer is more frequently performed in elderly patients. However, the evidence for beneficial outcomes of pancreatic cancer resection in elderly is not straightforward.^1^ Moreover, the impact of age as an independent predictor for short- and long-term postoperative outcomes remains controversial.^2^ Previous studies on this subject concerned mainly single-center studies with selected patients and small study groups, and without correction for frailty.^2,3^ A reflection of daily clinical practice on a nationwide scale with regard to this subject is lacking.

## Present

In this nationwide, prospective patient cohort, short- and long-term outcomes were compared between 198 patients aged ≥ 75 years and 638 patients aged < 75 years who underwent pancreatoduodenectomy for pancreatic cancer between 2014 to 2016.^4^ Following recent advancements in pancreatic cancer care, the surgical risk of performing pancreatic cancer surgery in patients aged ≥ 75 years appeared to be comparable with that of younger patients (major complications 31% vs. 28%; *P* = 0.43 and 90-day mortality 8% vs. 5%; *P* = 0.18, respectively). Nevertheless, elderly patients less often received adjuvant chemotherapy (37% vs. 69%; *P* < 0.001), and their overall survival was shorter (15 vs. 21 months; *P* < 0.001). Age ≥ 75 years was not independently associated with overall survival when adjusted for available frailty characteristics (i.e., polypharmacy, preoperative anemia, decreased renal function, Charlson Comorbidity Index ≥ 2, body mass index < 18.5 or ≥ 31, and the American Society of Anaesthesiologists score ≥ 3).

## Future

Results from this nationwide, multicenter, cohort study may contribute to the shared-decision making process for pancreatic cancer treatment in elderly patients. These results, reflecting real-world data, may be helpful to inform elderly patients on their surgical risk and emphasize on the importance of systemic therapy. However, it is essential to gain more insight in reasons to refrain from adjuvant chemotherapy, especially for elderly patients, in future prospective research. To increase the likelihood of receiving adequate chemotherapy and improve long-term oncological outcomes, frail elderly patients may benefit from reduced-dose chemotherapy, the use of modified 5-fluorouracil, leucovorin, irinotecan, oxaliplatin chemotherapy (FOLFIRINOX), or neoadjuvant chemotherapy.^5,6^ Future studies should address these questions.
